# Characteristics and outcomes associated with fidelity in the Family-Nurse Partnership in England: a data linkage cohort study

**DOI:** 10.1136/archdischild-2024-327654

**Published:** 2025-01-29

**Authors:** Amanda Clery, Francesca Cavallaro, Eilis Kennedy, Ruth Gilbert, Katie L Harron

**Affiliations:** 1 Great Ormond Street Institute of Child Health, UCL, London, UK; 2 The Health Foundation, London, UK; 3 Tavistock and Portman NHS Foundation Trust, London, UK

**Keywords:** Adolescent Health, Child Health Services, Epidemiology, Health services research

## Abstract

**Objectives:**

To determine (1) which maternal and area characteristics are associated with reaching fidelity targets (the expected number of visits mothers should receive at each stage of the programme) in the Family-Nurse Partnership (FNP), and (2) whether achieving these fidelity targets affects outcomes.

**Design, setting and population:**

Cohort study of mothers enrolled in the FNP, aged 13–19 years, giving birth between April 2010 and January 2018 in England. Mothers were linked to their Hospital Episode Statistics and National Pupil Database records.

**Outcomes:**

We described whether mothers reached fidelity targets for each programme stage (pregnancy, infancy and toddlerhood) and explored the characteristics associated with reaching targets. We used generalised linear models to compare child and maternal outcomes between mothers who did and did not reach fidelity targets.

**Results:**

Of the 28 155 mothers enrolled, 58% completed the programme. Fidelity targets were met by 59% of mothers in pregnancy, 65% in infancy and 61% in toddlerhood. The median number of visits was 38 (median 43 hours contact time). Younger mothers, those with a history of unplanned hospital admissions for adversity and those with social care involvement received a greater number of visits. Meeting fidelity targets was associated with a reduction in subsequent births within 18 months and an increase in the number of children with unplanned hospital admissions for maltreatment or injury up to age 2.

**Conclusions:**

Achieving fidelity to the FNP is challenging, but family nurses are able to engage the most vulnerable mothers in the programme. More research is needed to understand whether fidelity to programme targets is a useful measure of mothers’ experiences of intensive home visiting.

**WHAT IS ALREADY KNOWN ON THIS TOPIC**

- There is evidence to suggest that the Family-Nurse Partnership (FNP), an intensive home visiting service for young mothers, can be implemented with fidelity in a United Kingdom context.
- There is a lack of evidence on whether fidelity to the high number of visits in the programme schedule has any impact on maternal and child outcomes.

**WHAT THIS STUDY ADDS**

- Using linked, population-level data, this study explored the characteristics associated with fidelity to the FNP, and whether fidelity is associated with outcomes, in more than 28 000 mothers enrolled in the programme between 2010 and 2018.
- We found that only around 60% of mothers were meeting the expected number of visits, although the youngest mothers and those with some adversity indicators tended to receive more visits.
- Meeting fidelity targets at different stages of the programme was associated with some maternal and child health outcomes, including a reduction in subsequent births within 18 months, and an increase in unplanned hospital admissions for maltreatment or injury for mothers’ children up to age 2.

**HOW THIS STUDY MIGHT AFFECT RESEARCH, POLICY OR PRACTICE**

- Low fidelity is not always an indicator of a negative outcome: more research with mothers and their family nurses is needed to understand whether requiring fidelity to the schedule of visits is appropriate or whether other indicators better reflect engagement.
- Evidence on the barriers and enablers of engagement with the programme could be used to better deliver and commission intensive home visiting services.

## Introduction

 The Family-Nurse Partnership (FNP) is an intensive public health programme for young, first-time mothers.^1^ It is delivered from early pregnancy until the child is 2 years old by family nurses, aimed at improving pregnancy outcomes, child health and development, and parents’ economic self-sufficiency.^2^,^4^ The FNP was first developed in the USA in the 1980s-1990s and trialled in England in 2009–2010 (The Building Blocks Trial).^2 5^ Since then, it has been delivered in over 130 local authorities (LAs) across England.^6 7^ Mothers are invited to enrol in the programme during pregnancy if they are aged <20 (<25 in some areas) and nulliparous; however, due to limited places and its voluntary nature, only around a quarter of eligible mothers are enrolled.^7^

The FNP is a licensed programme, and as part of the terms, delivery must meet core requirements to ensure maximum benefit, including that mothers receive a majority of the scheduled visits (fidelity to the programme).^1^ There is a schedule of 64 visits across three programme stages: 14 visits in the pregnancy stage (from 16 weeks gestation), 28 in infancy (birth until the child’s first birthday) and 22 in toddlerhood (between one and 2 years old).^8^

A 2010 implementation evaluation found that the FNP can be implemented with fidelity in the UK.^9^ Original evidence from the trials in the USA painted a mixed picture for whether higher visit attendance improved outcomes.^10^ The Building Blocks Trial also found that variation in the number of visits did not affect outcomes. Therefore, there is currently no clear evidence for an impact of high fidelity to the programme schedule on programme effectiveness.

Qualitative research with family nurses has described challenges with fidelity targets, including providing more tailored support than the schedule of visits allows. For example, some participants described that not all visits were necessary when mothers were supported by other services.^8 11^ Furthermore, the resource required to deliver the large volume of visits means that the FNP is a highly rationed and selective service.^7 12^ Understanding the barriers and enablers of fidelity is key to improve engagement with the programme, interpret differences in the findings across trials and help policymakers plan and commission intensive home visiting services that provide flexibility and meet population needs.

We used linked longitudinal data for all women enrolled in FNP in England between 2010 and 2018 to (1) determine which maternal and area characteristics were associated with reaching fidelity targets, and (2) assess whether achieving these targets had any effect on selected outcomes.

## Methods

### Data source and population

We used data from the FNP National Unit on all mothers enrolled in the programme who gave birth between April 2010 and January 2018, followed up until January 2020 (allowing mothers to complete the 2-year programme). Mothers were linked to their Hospital Episode Statistics (HES) and their education and social care records (the National Pupil Database; NPD). Full details for creation of the cohort are described elsewhere.^13^

### Description of fidelity targets

The FNP licence outlines fidelity targets for the expected number of visits mothers should receive at each stage of the programme (table 1). In this study, we only expected mothers to receive visits when active in the programme. We described programme fidelity targets for mothers by calculating the proportion of visits completed out of the expected number of visits, according to actual time spent in the programme. For example, a mother who left early (because they were returning to work and no longer had time, or felt they would no longer benefit from visits), but who received all visits before leaving, was categorised as having 100% fidelity. In a small number of cases, mothers had interrupted periods of enrolment, where they left the programme and returned later (online supplemental file 1). We also described programme attrition (overall target of <40%), mean visit length (target of 1 hour per visit) and total time spent in the programme for all enrolled mothers.^8^

**Table 1:** Fidelity targets: the number and percentage of visits mothers are expected to receive for each stage of the Family-Nurse Partnership

Programme stage	Expected visits	Maximum possible number of visits	Fidelity target (percentage of visits)	Attrition target(%)
Pregnancy	One hour, weekly for the first 4 weeks, then every 2 weeks until birth	14 for those enrolled from 16 weeks gestation	80%(at least 11 visits for full stage)	<10
Infancy(up to the child’s first birthday)	One hour, weekly for the first 6 weeks, then every 2 weeks	28	65%(at least 18 visits for full stage)	<20
Toddlerhood(child aged 1–2 years)	One hour, every 2 weeks for the first 10 months, then monthly	22	60%(at least 13 visits for full stage)	<10

### Objective 1: characteristics associated with fidelity targets

We described maternal and area characteristics associated with fidelity. We selected characteristics that indicate vulnerability and might be associated both with engagement in the programme or with increased risk, from four sources: (1) FNP records, (2) hospital records (HES), (3) education and social care records (NPD) and (4) publicly available aggregate data (online supplemental file 2). To estimate the association between each of the characteristics in online supplemental file 2 and engagement, we fitted separate models (with appropriate confounders, informed by Directed Acyclic Graphs) and present coefficients from each of these models in the same table (avoiding the table 2 fallacy) (Westreich and Greenland^14^). We categorised maternal age as we did not expect a linear relationship with each year of age to align with previous literature on the FNP, and to align with how practitioners choose how to prioritise who to enrol in the programme.

**Table 2:** Summary of time and visit targets in each stage of the Family-Nurse Partnership programme for mothers giving birth between April 2010 and January 2018 aged 13–19 at last menstrual period

	Full programme	Pregnancy	Infancy	Toddlerhood
Number of mothers in each stage	28 155	28 155	25 975	20 015
Attrition* (%)	11 850 (42.1)	2180 (7.7)	5910 (22.8)	3760 (18.8)
Number of mothers who were in each stage for >2 weeks	28 155	28 155	25 905	19 990
Mean time in the stage (SD), weeks	94.9 (41.3)	20.3 (6.4)	46.0 (13.1)	45.2 (14.6)
Median time in the stage (IQR), weeks	118 (63–128)	21 (16–25)	52 (52–52)	52 (46–53)
Mean number of visits (SD)	35.9 (17.0)	9.8 (3.6)	10.3 (3.3)	13.3 (6.5)
Median number of visits (IQR)	38 (24–49)	10 (8–12)	10 (8–13)	14 (9–18)
N that met target† (%)	7745 (27.5)	16 695 (59.2)	16 945 (65.4)	12 115 (60.6)
Number of mothers with duration of visit recorded	28 120	28 085	25 635	19 695
Mean visit length (SD), hours‡	1.2 (0.2)	1.2 (0.2)	1.1 (0.2)	1.1 (0.2)
Median visit length (IQR), hours‡	1.0 (1.0–1.3)	1.2 (1.0–1.5)	1.1 (1.0–1.2)	1.0 (1.0–1.2)
Mean total time spent with a nurse (SD), hours	42.1 (21.4)	12.1 (5.1)	21.5 (9.0)	15.4 (8.0)
Median total time spent with a nurse (IQR), hours	43 (27–57)	12 (9–15)	22 (16–27)	15 (10–20)

Counts have been rounded to the nearest 5, in accordance with NHS England’s guidance on statistical disclosure control.

See online supplemental file 1 for details of flow of participants through each programme stage.

* Compared with a target of <10% during pregnancy, <20% during infancy and <10% during toddlerhood

† Compared with a target of 80% during pregnancy, 65% during infancy and 60% during toddlerhood. N for the full programme is the number of mothers meeting targets in all stages.

‡ Compared with a target of 1 hour per visit. These are the mean of the means, or medians of the medians (ie, mean number of visits for each mother, averaged across all mothers).

NHS, National Health Service.

We used multilevel, generalised linear models with mothers nested within FNP sites to explore the association between maternal/area characteristics and meeting fidelity targets. We fitted separate models for the three programme stages. We calculated relative risks (RRs) and CIs, adjusted for all other characteristics.

About 10.6% of mothers could not be linked to NPD and therefore did not have information on NPD variables (online supplemental file 1). Then, 3.3% of mothers had missing ethnicity, <0.5% had missing data on the percentage of visits where partner was present and 2.8% were missing data on Child in Need (CiN) status or Child Protection Plans (CPP) at enrolment. We included mothers who had missing data in the ethnicity and NPD variables in the analysis using an ‘unknown’/‘unlinked’ category, and excluded those who had missing data in any other variables. This approach should be unbiased under the assumption that the chance of being a complete case (ie, no missingness on CiN/CPP at enrolment, and percentage of visits with partner/parent present) does not depend on the outcome (eg, fidelity) after taking the other covariates into consideration.^15^ As a sensitivity analysis, we ran multiple imputation with chained equations for missing data on CiN/CPP at enrolment, and percentage of visits with partner/parent present, with 25 imputed datasets.

### Objective 2: association between achieving fidelity targets and child/maternal outcomes

Our outcomes of interest were unplanned child admissions for maltreatment or injury up to age 2 (online supplemental file 4), a good level of development at age 5 (school readiness, from the NPD,^16^ unplanned maternal admissions for any diagnosis in the 2 years after birth and subsequent deliveries within 18 months of the index birth.

We used the same modelling approach as objective 1 to explore whether meeting fidelity targets was associated with these outcomes, adjusting for meeting the fidelity targets in previous programme stages, those who left the programme before reaching the relevant stage and for maternal/area characteristics (online supplemental file 1) expected to be related to both engagement and outcomes. We also explored whether the total number of visits received was associated with outcomes.

All values presented are rounded to the nearest 5 and all small numbers <7 are suppressed, in line with NHS England’s statistical disclosure rules.^17^

## Results

We included 28 155 mothers enrolled in the FNP aged 13–19 at last menstrual period, who had their first live birth between April 2010 and January 2018 and who were linked with HES.

### Description of fidelity targets

**Figure 1:**
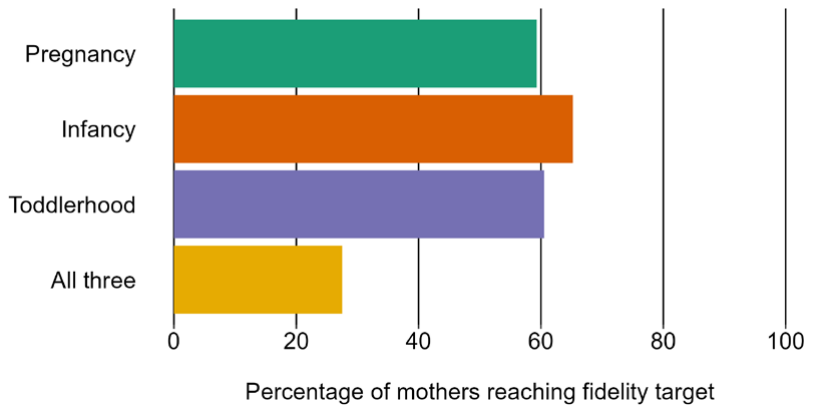
Percentage of mothers reaching visit fidelity targets* in each stage of the Family-Nurse Partnership for mothers giving birth between April 2010 and January 2018 aged 13–19 at last menstrual period. *Fidelity targets=80% of visits (n=11) in pregnancy, 65% of visits (n=18) in infancy and 60% of visits (n=13) in toddlerhood.

Of the 28 155 mothers included, 28 120 had at least one visit recorded; 16 305 (58%) completed the programme, and overall attrition was 42%. Only 28% of mothers met the fidelity targets across all three stages of the programme (figure 1). Mothers received a median of 38 out of 64 possible visits, corresponding to a median of 43 hours contact time with a family nurse (out of the recommend 64 hours). Mean visit length was 1.2 hours (median 1.0 hours) across the programme.

The majority of FNP mothers (25 925; 92%) completed the pregnancy stage in full. The median number of visits during pregnancy was 10 out of 14 possible visits; 16 695 mothers (59%) met the fidelity target of 80% (n=11 visits).

Of the 25 975 mothers in the programme at the infancy stage, 19 860 (77%) completed the stage. The median number of visits received was 10 out of 28 possible visits; 16 945 mothers (65%) met the 65% fidelity target of 65% (n=18 visits).

Of the 20 015 mothers in the programme at the toddlerhood stage, 16 175 (81%) completed the stage. The median number of visits received was 14 out of 22 possible visits; 12 115 mothers (61%) met the 60% fidelity target (n=13 visits).

### Characteristics associated with fidelity targets

There were 27 360 mothers with complete data on characteristics who started the pregnancy stage, and 25 635 and 19 655 for the infancy and toddlerhood stages, respectively. The greatest driver of meeting fidelity targets during the infancy and toddlerhood stages was having met the targets in the previous stages of the programme. Mothers who met the pregnancy target were 50% more likely to meet the infancy target compared with those who had not (RR 1.56; 95% CI 1.50, 1.61). Mothers who met the infancy target were almost twice as likely to meet the toddlerhood target compared with those who had not (RR 1.98; 95% CI 1.90, 2.07).

**Figure 2:**
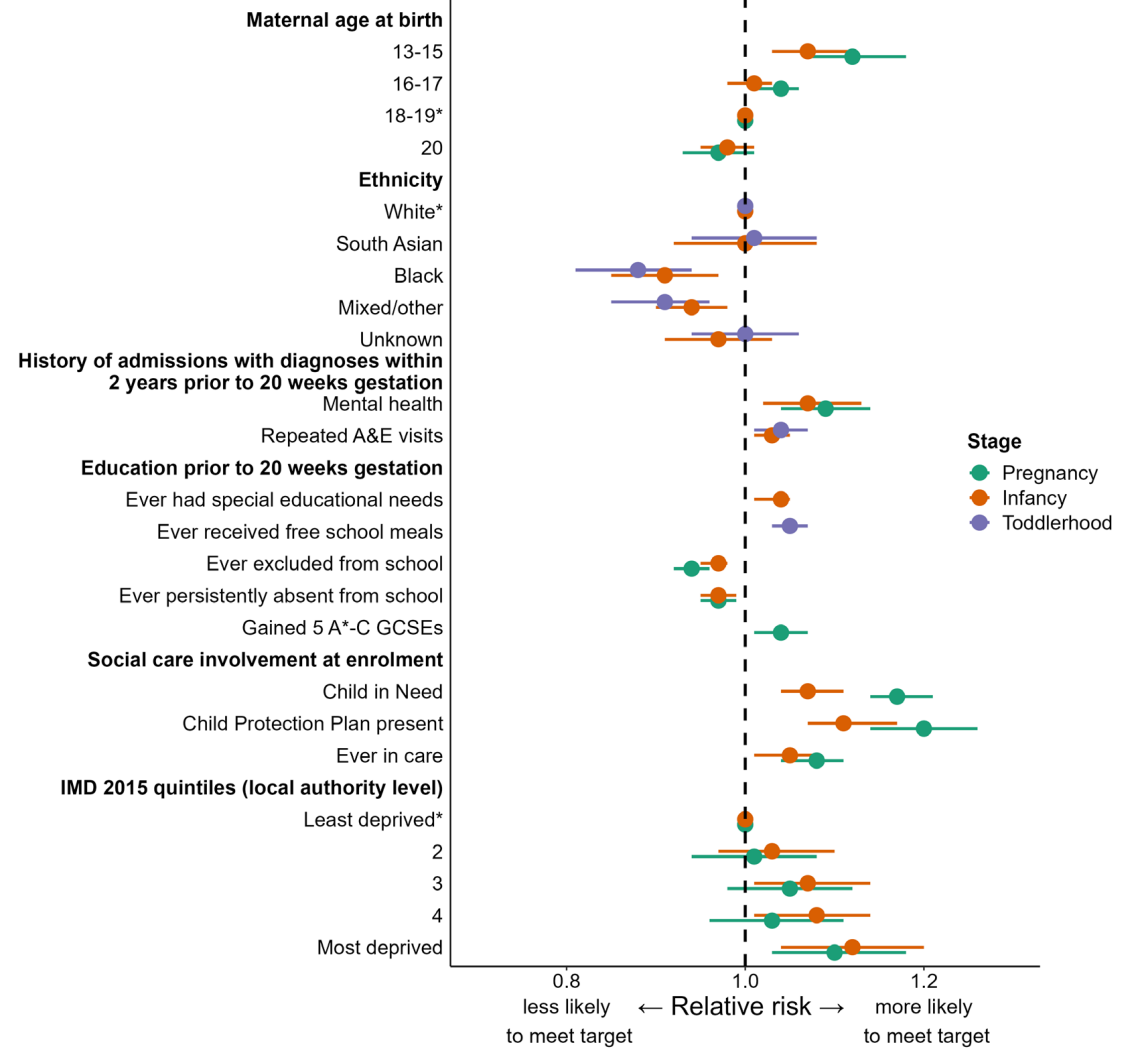
Maternal and area characteristics associated with reaching fidelity targets in each stage of the Family-Nurse Partnership programme for mothers giving birth between April 2010 and January 2018 aged 13–19 at last menstrual period. *Indicates reference group. If not stated, reference group is those who did not have the characteristic described. Points represent effect estimates. Horizontal bars represent 95% CIs. If an association is not shown with a characteristic at a particular FNP stage, that characteristic was not statistically significantly associated with fidelity. IMD, Index of Multiple Deprivation. Underlying data available in online supplemental file 5.

Different maternal and area characteristics were associated with reaching fidelity targets at each programme stage (figure 2, online supplemental file 5). In adjusted models, the greatest drivers of meeting fidelity targets in the pregnancy stage were young maternal age (RR 1.12; 95% CI 1.07, 1.17 for mothers aged 13–15 compared with 18–19), having a history of hospital admissions for mental health (RR 1.09; 95% CI 1.04, 1.14 compared with mothers without a history) and being a child in need or having a child protection plan at enrolment (RR 1.17; 95% CI 1.14, 1.21 and RR 1.20; 95% CI 1.14, 1.26, respectively, compared with mothers who did not). Results were almost identical in the sensitivity analysis using multiple imputation (online supplemental file 6). In the pregnancy and toddlerhood stages, mothers who had ever been recorded with persistent absence from school were less likely to meet targets than those who had not, and those who had achieved 5 A*-C General Certificates of Secondary Education (GCSEs) prior to enrolment were more likely to meet the pregnancy target than those who had not. Ethnicity was associated with meeting the infancy and toddlerhood targets (RR 0.91; 95% CI 0.85, 0.97 and RR 0.88; 95% CI 0.81, 0.94 for black mothers compared with white, respectively).

Index of Multiple Deprivation (IMD) at the FNP area level was associated with fidelity in the pregnancy and infancy stages, with mothers in the most deprived areas being more likely to meet targets than those in the least deprived areas (RR 1.10; 95% CI 1.03, 1.18). There was no association between fidelity targets and the percentage of eligible mothers enrolled in the area.

### Effect of fidelity targets on child and maternal outcomes

Meeting the fidelity target in pregnancy was associated with a reduction in subsequent births within 18 months (RR 0.87; 95% CI 0.78, 0.97 compared with mothers who did not meet the target; table 3). Visit fidelity in infancy and toddlerhood was associated with an increase in the number of children with unplanned hospital admissions for maltreatment or injury up to age 2 (table 3). A similar pattern was seen according to total number of visits.

**Table 3:** Adjusted relative risks and 95% CIs comparing child and maternal outcomes according to reaching fidelity targets at each stage of the Family-Nurse Partnership programme, and total number of visits, for mothers giving birth between April 2010 and January 2018 aged 13–19 at last menstrual period

	Child unplanned hospital admissions for maltreatment/injury up to age 2	Good level of development (school readiness)	Maternal unplanned admissions for any diagnosis in the 2 years following birth	Subsequent live births within 18 months of the index child
	Relative risk*(**95% CI**)	Relative risk*(**95% CI**)	Relative risk*(**95% CI**)	Relative risk* (95% CI)
Met pregnancy fidelity target (80%)	0.90 (0.90, 1.03)	0.97 (0.92, 1.02)	1.00 (0.94, 1.06)	0.87 (0.78, 0.97)
Met infancy fidelity target (65%)	1.27 (1.12, 1.43)	0.98 (0.93, 1.04)	1.02 (0.94, 1.10)	0.90 (0.79, 1.03)
Met toddlerhood fidelity target (60%)	1.21 (1.09, 1.35)	0.98 (0.92, 1.04)	1.00 (0.94, 1.07)	0.97 (0.82, 1.14)
Number of visits in pregnancy	0.99 (0.98 to 1.01)	1.01 (1.00 to 1.01)	1.00 (0.99 to 1.01)	0.96 (0.94 to 0.98)
Number of visits in infancy	1.01 (1.00 to 1.02)	1.00 (0.99 to 1.00)	1.00 (1.00 to 1.01)	1.00 (0.98 to 1.01)
Number of visits in toddlerhood	1.00 (1.00 to 1.01)	1.00 (1.00 to 1.00)	1.00 (0.99 to 1.00)	0.99 (0.98 to 1.00)

* Relative risks are adjusted for all covariates associated with meeting the target in each stage of the programme and with outcomes.

## Discussion

Of the 28 155 mothers enrolled in FNP and included in our study, 58% completed the programme, 60–65% met the fidelity target for any one stage and only 28% met the fidelity targets for all three programme stages. Mothers received an average of 38 visits (10 in pregnancy, 10 in infancy and 14 in toddlerhood), corresponding to an average of 43 hours contact time with a family nurse, indicating that visits were generally longer than the recommended 1 hour.

These findings suggest a significant gap between the FNP programme theory and the ‘real world’ implementation of FNP in an English context. Despite lower levels of engagement than considered necessary overall, the most vulnerable and youngest mothers tended to be more likely to meet the fidelity targets than those who did not have these risk factors. This may suggest that family nurses are able to make decisions about where to prioritise their time and resources to provide more intensive support to those they feel most needed it, or that these mothers found the programme more useful or were under more pressure to engage than others.

Interpreting the high attrition rate, and why fidelity targets are not met, is complex. Mothers may choose to leave the programme because they feel they have received the support they need, indicating that attrition may not in itself be a negative outcome, as has been found in other settings.^18^ After the publication of the Building Blocks Trial results and qualitative research suggesting that mothers wanted more flexibility within the programme, the FNP National Unit decided to allow greater flexibility in the visit schedule and programme graduation, now decided locally.^19 20^ Further exploration with nurses and mothers to understand whether number of visits received is an appropriate fidelity target, or if personalising the intervention according to need would be of benefit to both FNP delivery and commissioning.

We found that mothers who met the pregnancy visit fidelity target were less likely to have a subsequent birth within 18 months than those who did not; children born to mothers who met the target in infancy and toddlerhood were more likely to have an unplanned hospital admission for maltreatment or injury up to age 2 than those who did not meet the targets. Interpretation of these findings is not straightforward, since the most vulnerable mothers receive more visits but are also independently more likely to experience worse outcomes. However, our findings mirror those of previous research showing similar associations between enrolment in FNP and unplanned hospital admissions for maltreatment or injury up to age 2 and subsequent pregnancy within 18 months.^21^ Previous research has also suggested that interventions with fewer sessions and a narrow focus are more effective at enhancing parental sensitivity and promoting attachment, and that highly intensive interventions may even be detrimental.^22^ These findings, in combination with a context of a highly targeted programme, warrants further exploration of why mothers leave the programme early.

### Strengths and limitations

A major strength of our study is the use of administrative data, allowing our analysis to include all mothers enrolled in the FNP over an 8-year period. Another strength is the use of data linkage to obtain the hospital, education and social care records for FNP mothers, which enabled us to use additional, objective measures of maternal characteristics collected outside of those recorded by FNP nurses.

A further strength is that our results are similar to those reported in the Building Blocks Trial, where mothers received an average 39 visits (10, 19 and 13 during the three stages).^2 8^ We observed slightly higher fidelity than reported in the Buildings Blocks Trial, which observed 58% in pregnancy, 53% in infancy and 44% in toddlerhood.^8^ This may be due to differences in the way that the expected number of visits was calculated between studies, as we took into account the length of time for which a mother was enrolled. Our findings also support earlier research that showed family nurses are able to target enrolment in the FNP to the more vulnerable mothers.^7^

The main limitation of our analyses was that we were restricted by data routinely collected by FNP nurses and other services, and hence the quality of the recording of these data. It is unlikely that we captured information on all relevant maternal characteristics associated with fidelity, particularly those less severe vulnerabilities than would have resulted in hospital attendances or involvement with social care. Residual confounding in the relationship between fidelity targets and outcomes may therefore be present. Our analysis was further limited by missing data on NPD variables for around 10% of mothers, and lower levels of missing data on ethnicity, CiN/CPP at enrolment, and the percentage of visits where the partner was present. Our approach to handling missing data may have led to bias if these values were missing not at random.

## Conclusion

Achieving and maintaining high levels of fidelity to intensive home visiting programmes is challenging for young mothers and their family nurses. However, family nurses appear to be able to encourage engagement for the most vulnerable mothers, including those with a history of hospital attendances and involvement with social care. More research is needed to understand whether fidelity to programme targets is a useful measure of mothers’ experiences of intensive home visiting.

## Supplementary material

10.1136/archdischild-2024-327654 online supplemental file 1

## Data Availability

Data may be obtained from a third party and are not publicly available.
