# Supplementary material for: Characteristics and outcomes associated with fidelity in the Family-Nurse Partnership in England: a data linkage cohort study
Source: Arch Dis Child. 2025 Jan 29;110(7):e327654. doi: 10.1136/archdischild-2024-327654 (PMC12229051; doi:10.1136/archdischild-2024-327654)
Supplement: online supplemental file 1 [file archdischild-110-7-s001.pdf]

Characteristics and outcomes associated with fidelity in the Family Nurse Partnership in England: a data linkage cohort study

Supplementary Files

Supplementary File 1: Flow of participants through the different stages of the FNP programme

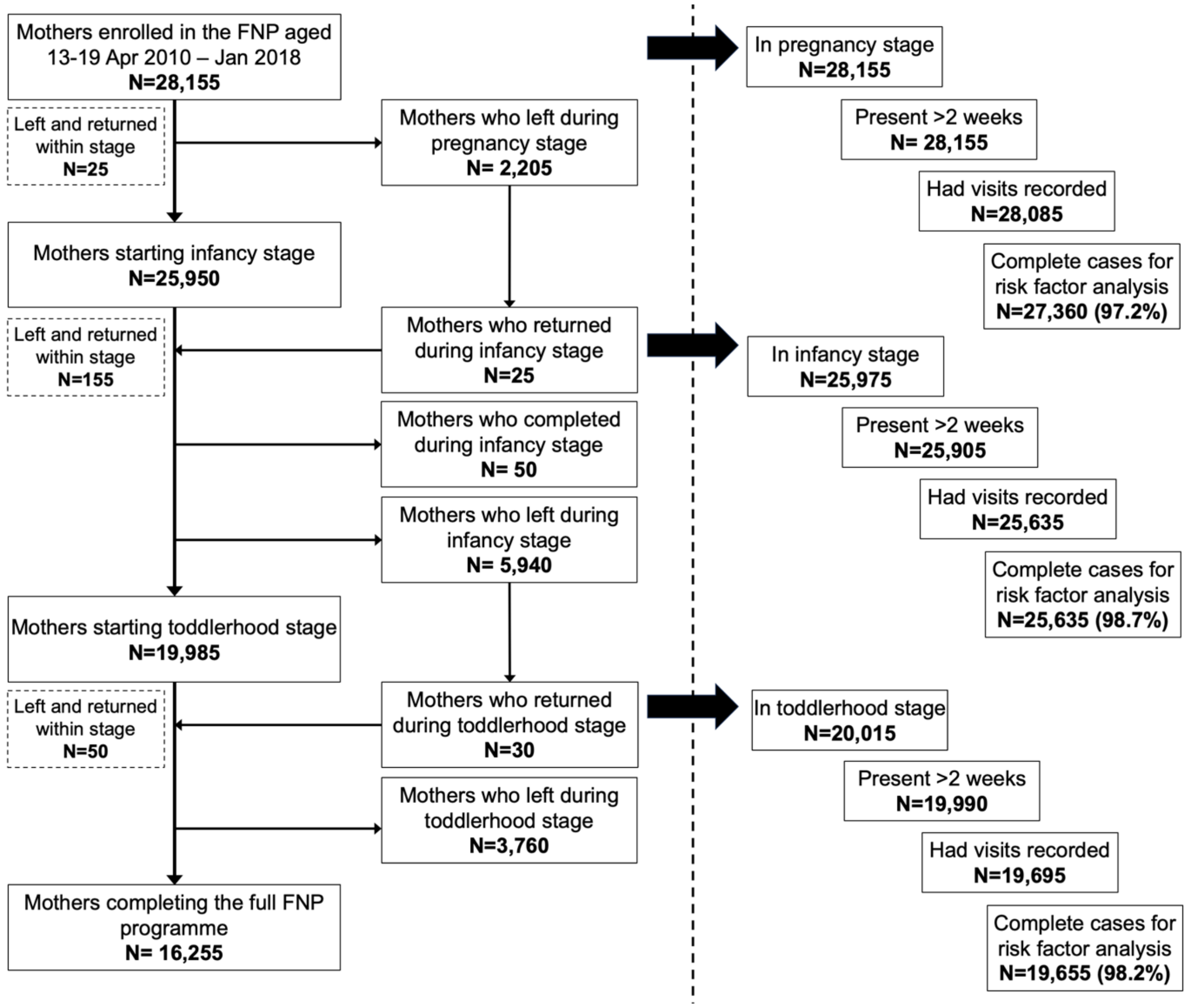

Supplementary File 2: List of characteristics used for exploration of fidelity targets

Hospital Episode Statistics (maternal)

- Maternal age
- Ethnicity [White, South Asian, Black, Mixed/other, unknown]
- Neighbourhood-level deprivation [Index of Multiple Deprivation; IMD quintile]
- Region of residence [North East, North West, Yorkshire and Humber, East Midlands, West Midlands, East of England, London, South East, South West]
- Gestational age at booking
- History of unplanned hospital admissions in the two years prior to 20 weeks gestation for adversity-related, or mental health-related diagnoses, any admission for chronic condition-related diagnoses, based on ICD-10 diagnosis codes (supplemental file 3)
- A&E attendances [any or repeat (4+) attendances] in the two years prior to 20 weeks gestation
- Outpatient attendances [no outpatient referrals, attended, did not attend]

National Pupil Database (maternal)

- Ever in care or had a child protection plan recorded prior to 20 weeks gestation
- Ever recorded as having special educational needs provision
- Ever recorded as having free school meals
- Ever in the bottom Income Deprivation Affecting Children Index (IDACI) decile
- Ever excluded from school, in a pupil referral unit or alternative provision
- Ever recorded as persistently absent (absent for ≥10% of possible school sessions) in a term
- Educational attainment measured by General Certificate of Secondary Education (GCSEs) gained [achieved 5 A*-C GCSEs or not, did not attempt GCSEs (too young)]

Family Nurse Partnership (maternal)

- Primary language [English or other]
- Living arrangements [alone, foster care, with mother, with partner, with other adults]
- Marital status [married, cohabiting, separated, single]
- Number of benefits received
- Alcohol or drug use in the 2 weeks prior to enrolment
- Child in need status or child protection plan at enrolment
- Percentage of visits for which a partner or parent was present

Publicly available aggregate data (FNP site)

- LA-level IMD quintile
- Rural-urban classification [predominantly rural, predominantly urban, urban with significant rural]
- Percentage of mothers in the LA who are teenagers
- Percentage of term live births with low birth weight
- Rate of premature births
- Ethnic distribution in the population
- Rate of children on child protection plans
- Rate of referrals to children's social care
- Rate of enrolment of eligible mothers into the FNP

Supplementary File 3: ICD-10 codes for maternal characteristics

ICD-10 code lists for maternal hospital admissions related to adversity, mental health, and chronic conditions. Code lists were derived from the following studies:

- Harron K, Gilbert R, Fagg J, Guttmann A, van der Meulen J. Associations between pre-pregnancy psychosocial risk factors and infant outcomes: a population-based cohort study in England. The Lancet Public Health 2021; 6(2): e97-e105.
- Herbert A, Gilbert R, González-Izquierdo A, et al. Violence, self-harm and drug or alcohol misuse in adolescents admitted to hospitals in England for injury: a retrospective cohort study. BMJ Open. 2015;5(2):e006079.
- Hardelid P, Dattani N, Gilbert R. Estimating the prevalence of chronic conditions in children who die in England, Scotland and Wales: a data linkage cohort study. BMJ Open. 2014;4(8).
- Pearson RJ, Jay MA, Wijlaars LPMM, et al. Association between health indicators of maternal adversity and the rate of infant entry to local authority care in England: a longitudinal ecological study. BMJ Open 2020; 10(8): e036564.

Unplanned maternal hospital admissions with any of the ICD-10 codes in the following respective categories were considered to be adversity-related or mental health-related. Mothers with any hospital admissions relating to a chronic condition were considered to have a chronic condition.

Group	Description	ICD10 Code
**Adversity-related admissions**		
**Violence**	Maltreatment syndromes	T74
	Effects of other deprivation (extreme neglect)	T73
	Perpetrator of neglect and other maltreatment syndromes	Y06, Y07
	Assault by bodily force and sexual assault	Y04, Y05
	Other types of assault	X85-Y03, Y08-Y09
	Events of undetermined intent	Y20-Y34
	Examination and observation following other inflicted injury	Z04.5
	Examination and observation for other reasons: request for expert evidence	Z04.8
**Self-harm**	Sequelae of intentional self-harm	Y87.0
	Intentional self-poisoning by and exposure to … drugs	X60-X63
	…other and unspecified drugs, medicaments and biological substances	X64
	…alcohol	X65
	…organic solvents and halogenated hydrocarbons and their vapours	X66
	…other gases and vapours	X67
	…pesticides	X68
	…other and unspecified chemicals and noxious substances	X69
	Intentional self-harm by… hanging, strangulation and suffocation	X70
	…drowning and submersion	X71

	…firearm discharge	X72-X74
	…explosive material	X75
	…smoke, fire and flames, or steam, hot vapours and hot objects	X76-X77
	…sharp/blunt objects	X78-X79
	...jumping from a high place	X80
	…jumping or lying before a moving object, or crashing a motor vehicle	X81-82
	…other specified means	X83
	…unspecified means	X84
**Substance misuse**	Mental and behavioural disorders due to psychoactive substance use	F11-F16, F19
	Finding of drugs not normally found in blood	R78.1-R78.5
	Poisoning by drugs, medicaments and biological substances	T36-T50 (not T50.6)
	Poisoning, undetermined intent	Y10-Y14
	Drug rehabilitation	Z50.3
	Drug abuse counselling and surveillance	Z71.5
	Drug use	Z72.2
	Mental and behavioural disorders due to use of volatile solvents	F18
	Accidental poisoning by and exposure to noxious substances	X40–X44, X46-X49
	Poisoning by chemical or noxious substance, undetermined intent	Y16-Y19
	Special epileptic syndromes - (related to alcohol, drugs, etc.)	G40.5
	Blood-alcohol and blood-drug test	Z04.0
	Alcohol-induced pseudo-Cushing's syndrome	E24.4
	Mental and behavioural disorders due to use of alcohol	F10
	Degeneration of nervous system due to alcohol	G31.2
	Alcoholic polyneuropathy	G62.1
	Alcoholic myopathy	G72.1
	Alcoholic cardiomyopathy	I42.6
	Alcoholic gastritis	K29.2
	Alcoholic liver disease	K70
	Alcohol-induced acute pancreatitis	K85.2
	Alcohol-induced chronic pancreatitis	K86.0
	Maternal care for (suspected) damage to fetus from alcohol	O35.4
	Finding of alcohol in blood	R78.0
	Poisoning: antidotes and chelating agents, not elsewhere classified	T50.6
	Toxic effect of alcohol	T51
	Accidental poisoning by exposure to alcohol	X45
	Poisoning by exposure to alcohol, undetermined intent	Y15
	Evidence of alcohol involvement determined by blood alcohol level	Y90

	Evidence of alcohol involvement determined by level of intoxication	Y91
	Alcohol rehabilitation	Z50.2
	Alcohol abuse counselling and surveillance	Z71.4
	Alcohol use	Z72.1
**Mental health conditions / behavioural disorders (excluding those falling under adversity or chronic conditions)**		
	Organic, including symptomatic, mental disorders	F00-F09*
	Schizophrenia, schizotypal and delusional disorders	F20-F29
	Mood [affective] disorders	F30-F39
	Neurotic, stress-related and somatoform disorders	F40-F48
	Behavioural syndromes associated with physiological disturbances and physical factors	F50-F59**
	Disorders of adult personality and behaviour	F60-F69***
	Mental retardation	F70-F79
	Disorders of psychological development	F80-F89
	Behavioural and emotional disorders with onset usually occurring in childhood and adolescence	F90-F98
	Sedatives, hypnotics and antianxiety drugs	Y47
	Psychotropic drugs, not elsewhere classified	Y49
**Chronic conditions (except those falling under mental health conditions)**		
**Cancer/blood disorders**	Neoplasms	C00-C97, D00-D02, D05-D09, D12, D13, D14.1-D14.4, D15, D20, D32-D35, D37- D48, D63.0, E34.0, E88.3, G13.0, G13.1, G53.3, G55.0, G63.1, G73.1, G73.2, G94.1, M36.0, M36.1, M49.5, M82.0, M90.6, M90.7, N08.1, N16.1, Y43.1-Y43.3, Y84.2, Z08, Z51.0-Z51.2, Z54.1, Z54.2, Z85, Z86.0, Z92.3
	Immunological disorders	D80-D84, G53.2, Q98.0
	Anaemia and other blood disorders	D50, D56.0-D56.2, D56.4, D56.8, D56.9, D57.0-D57.2, D57.8, D58, D61.0, D61.9, D64, D66, D67, D68.0-D68.2, D68.4-D68.9, D69, D70-D76, M36.2-M36.4, M90.4, N08.2, Z86.2
**Chronic infections**	HIV	B20-B24, F02.4, R75, Z21
	Tuberculosis	A15-A19, E35.0, K23.0, K67.3, K93.0, M01.1, M49.0, P37.0
	Other	A50, A81, B18, B37.1, B37.5, B37.6, B37.7, B38.1, B39.1, B40.1, B44.0, B44.7, B45, B46, B48.7, B50.0, B50.8, B51.0, B51.8, B52.8, B52.0, B55, B57.2-B57.5, B58.0, B59, B67, B69, B73, B74, B78.7, B90-B94, F02.1, K23.1, K93.1, M00, N33.0, P35.0- P35.2, P35.8, P35.9, P37.1
**Respiratory**	Asthma and chronic lower respiratory disease	J41-J47

	Cystic fibrosis	E84, P75
	Injuries	S17, S27, S28, T27, T91.4
	Congenital anomalies	Q30-Q37, Q79.0
	Other	G47.3, J60-J70, J80-J86, J96.1, J98, P27, Y55.6, Z43.0, Z93.0, Z94.2
**Metabolic/endocrine /digestive /renal /genitourinary**	Diabetes	E10-E14, G59.0, G63.2, I79.2, M14.2, N08.3, O24, Y42.3
	Other endocrine	E00, E03.0, E03.1, E07.1, E22.0, E23.0, E25, E26.8, E29.1, E31, E34.1, E34.2, E34.5, E34.8, G13.2, G73.5, Y42.1
	Digestive	K20, K21.0, K22, K23.8, K25-K28, K29.0, K29.1, K29.3-K29.9, K31, K50-K52, K55, K57, K59.2, K63.0-K63.3, K66, K72-K76, K80-K83, K85.0, K85.1, K85.8, K85.9, K86.1-K86.9, K87.0, K90, M07.4, M07.5, M09.1, M09.2, T86.4, Z43.2-Z43.4, Z46.5, Z90.3, Z90.4, Z93.2-Z93.5
	Renal/genitourinary	D63.8, G63.8, G99.8, I68.8, M90.8, N08.4, N00-N05, N07, N11-N15, N16.0, N16.2, N16.4, N16.5, N16.8, N18, N19, N20-N23, N25, N26, N28, N29, N31, N32, N33.8, N35, N36, N39.1, N39.3, N39.4, N40-N42, N70-N74, N80-N82, N85, N86, N87,N88, P96.0, T82.4, T83.1, T83.2, T83.4-T83.9, T85.5, T86.1, Y60.2, Y61.2, Y62.2, Y84.1, Z49, Z93.6, Z94.0, Z99.2
	Congenital anomalies of the digestive/renal/genitourinary system	Q38.0, Q38.3, Q38.4, Q38.6-Q38.8, Q39, Q40.2, Q40.3, Q40.8, Q40.9, Q41, Q42, Q43.1, Q43.3-Q43.7, Q43.9, Q44, Q45, Q50.0, Q51, Q52.0-Q52.2, Q52.4, Q54.0- Q54.3, Q54.8, Q54.9, Q55.0, Q55.5, Q56, Q60.1, Q60.2, Q60.4-Q60.6, Q61, Q62.0- Q62.6, Q62.8, Q63.0-Q63.2, Q63.8, Q63.9, Q64, Q79.2-Q79.5, Q87.8, Q89.1, Q89.2
	Injuries	S36, S37, S38, S39.6, S39.7, T06.5, T28, T91.5
	Other/unspecified	E66, G63.3, G99.0, M14.5, N92, Z86.3, Z93.8
**Musculoskeletal/ skin**	Musculoskeletal/connective tissue	G55.1-G55.3, G63.5, G63.6, G73.7, J99.0, J99.1, L62.0, M05, M06, M07.0-M07.3, M07.6, M08, M09.8, M10-M13, M14.0, M14.6, M14.8, M30-M35, M40-M43, M45-M48,M50-M54, M60-M62, M63.8, M80.1-M80.9, M81.1-M81.9, M82.1, M82.8, M84.0-M84.2, M84.8, M84.9, M85, M86.3-M86.6, M89, M90.0, M91-M94, N08.5, Y45.4

	Skeletal injuries/amputations	S13, S22.0-S22.2, S22.5, S23, S32, S33, S68.3, S68.4, S68.8, S77, S78, S87, S88, S97, S98.0, S98.2-S98.4, T02, T04, T05, T20.3, T20.7, T21.3, T21.7, T22.3, T22.7, T23.2, T23.3, T23.6, T23.7, T24.3, T24.7, T25.2, T25.3, T25.6, T25.7, T29.3, T29.7, T30.3, T30.7, T31.2-T31.9, T32.2-T32.9, T87.3-T87.6, T91.2 T91.8, T92.6, T93.1, T93.4, T93.6, T94.0, T94.1, T95.0, T95.1, T95.4, T95.8, T95.9, Y83.5, Z89.1, Z89.2, Z89.5-Z89.8, Z97.1
	Chronic skin disorders	L10, L11.0, L11.8, L11.9, L12-L14, L28, L40-L45, L57, L58.1, L59, L87, L88, L90, 3 L92, L95, L93, L98.5, M09.0, Q80, Q81, Q87.0-Q87.5, Q89.4
	Congenital anomalies	Q18.8, Q65.0-Q65.2, Q65.8, Q65.9, Q67.5, Q68.2, Q68.3-Q68.5, Q71-Q73, Q74, Q75.3-Q75.9, Q76.1-Q76.4, Q77, Q78, Q79.6, Q79.8, Q82.0-Q82.4, Q82.9, Q86.2, Q89.7-Q89.9
**Neurological**	Epilepsy	F80.3, G40.0-G40.4, G40.6-G40.9, G41, R56.8, Y46.0-Y46.6
	Cerebral palsy	G80-G83
	Injuries of brain, nerves, eyes or ears	S05-S08, S12, S14, S24, S34, S44, S54, S64, S74, S84, S94, T06.0-T06.2, T26, T90.4, T90.5, T91.1, T91.3, T92.4
	Chronic eye conditions	H05.1-H05.9, H13.3, H17, H18, H19.3, H19.8, H21, H26, H27, H28.0-H28.2, H31, H32.8, H33, H34, H35, H40, H42.0, H43, H44, H47, H54.0- H54.2, H54.4, T85.2, T85.3, Z44.2
	Chronic ear conditions	H60.2, H65.2-H65.4, H66.1-H66.3, H69.0, H70.1, H73.1, H74.0-H74.3, H75.0, H80, H81.0, H81.4, H83.0, H83.2, H90.0, H90.3, H90.5, H90.6, H91, Z45.3
	Perinatal conditions	P10, P21.0, P52, P57, P90, P91.1, P91.2, P91.6
	Congenital anomalies of neurological or sensory systems	Q00-Q07, Q10.4, Q10.7, Q11-Q12, Q13.0-Q13.4, Q13.8, Q13.9, Q14-Q16, Q75.0, Q75.1, Q85, Q86.0, Q86.1, Q86.8, Q90-Q93, Q95.2, Q95.3, Q97, Q99
	Other	F02.2, F02.3,G00-G09, G10-G12, G13.8, G14, G20-G23, G24.1-G24.9, G25-G30, G31.0-G31.1, G31.8, G31.9, G32-G37, G43-G46, G47.0-G47.2, G47.4-G47.9, G50- G52, G53.0, G53.1, G53.8, G54, G55.8, G56-G58, G59.8, G60, G61, G62.0,

		G62.2- G62.9, G64, G70, G71,G72.2-G72.9, G73.0, G73.3, G90-G93, G94.2, G94.8, G95, G96, G98, G99.1, G99.2, I60-I67, I68.0, I68.2, I69, I72.0, I72.5, T85.0, T85.1, Y46.7- Y46.8, Z98.2
**Cardiovascular**	Congenital heart disease	Q20-Q26, Q89.3
	Other	I00-I28, I31-I39, I41, I42.0-I42.5, I42.7-I42.9, I43.0, I43.1, I43.2-I43.8, I44.1-I44.7, I45.1-I45.9, I46-I51, I52.8, I70-I71, I72.1-I72.4, I72.8, I72.9, I73-I77, I79.0, I79.1, I79.8, I81-I82, I98-I99, M03.6, N08.8, Q27, Q28, S26, T82.0-T82.3, T82.5-T82.9, T86.2, Y60.5, Y61.5, Y62.5, Y84.0, Z45.0, Z50.0, Z94.1, Z95
**Codes indicating non-specific chronic conditions**	-	R62, R63.3, Z43.1, Z51.5, Z75.5, Z93.1, Z99.3

* Excluding F020-F024 (dementia codes)

** Excluding F51 (nonorganic sleep disorders) and F52 (sexual dysfunction, not caused by organic disorder or disease)

*** Excluding F64 (gender identity disorders (including transsexualism, transvestism, and “gender disorders”)

Supplementary File 4: ICD-10 codes for outcomes

ICD-10 code lists for child maltreatment and health care utilisation-related outcomes. Code lists were derived from the following studies:

- Gilbert R, Fluke J, O'Donnell M, et al. Child maltreatment: variation in trends and policies in six developed countries. Lancet 2011; 379 (9817):758-72
- Syed S, Ashwick R, Schlosser M, et al Predictive value of indicators for identifying child maltreatment and intimate partner violence in coded electronic health records: a systematic review and meta-analysis. Archives of Disease in Childhood 2021;106:44-53.
- Robling M, Lugg-Widger F, Cannings-John R, Sanders J, Angel L, Channon S, et al. The Family Nurse Partnership to reduce maltreatment and improve child health and development in young children: the BB:2–6 routine data-linkage follow-up to earlier RCT. Public Health Res 2021;9(2).

Unplanned hospital admissions with any of the specified ICD-10 codes were considered to be maltreatment or injury related.

Group	Description	ICD-10 Code	Age restriction
**Maltreatment and injury-related**			
**Injury & ingestion**	Injuries to the head (includes open wounds, fractures, crushing and dislocation)	S00-S09	N/A
	Injuries to the neck	S10-S19	N/A
	Injuries to the thorax	S20-S29	N/A
	Injuries to the abdomen, lower back, lumbar spine and pelvis	S30-S39	N/A
	Injuries to the shoulder and upper arm	S40-S49	N/A
	Injuries to the elbow and forearm	S50-S59	N/A
	Injuries to the wrist and hand	S60-S69	N/A
	Injuries to the hip and thigh	S70-S79	N/A
	Injuries to the knee and lower leg	S80-S89	N/A
	Injuries to the ankle and foot	S90-S99	N/A
	Injuries involving multiple body regions	T00-T07	N/A
	Injuries to unspecified part of trunk, limb or body region	T08-T14	N/A
	Effects of foreign body entering through natural orifice	T15-T19	N/A
	Burns and corrosions	T20-T32	N/A
	Frostbite	T33-T35	N/A
	Poisoning by drugs, medicaments and biological substances	T36-T50	N/A
	Toxic effects of substances chiefly non-medicinal as to source (sting, alcohol, solvents, etc.)	T51-T65	N/A
	Other and unspecified effects of external causes (effects of radiation, heat and light, hypothermia, electric shock, asphyxiation, food deprivation)	T66-T78	N/A
	Accidental poisoning by and exposure to noxious substances	X40-X49	N/A
**Maltreatment**	Maltreatment syndromes	T74	N/A
	Neglect and abandonment	Y06	N/A
	Other maltreatment	Y07	N/A

	Effects of other deprivation (hunger, thirst, exhaustion due to exposure or excessive exertion)	T73	N/A
	Assault	X85-Y05 Y08-Y09	N/A
**Maltreatment markers for infants <1yr**	Intracranial injuries	S06	<1yr
	Long-bone fractures	S42.2-S42.4, S42.7-S42.8, S52, S72, S82, T10, T12	<1yr
**Maltreatment markers for children ≤4yrs**	Traumatic brain injuries	S06, S09.7-S09.8, T90.5	≤4yrs
	Retinal haemorrhage	H35.6	≤4yrs
	Rib fractures	T29-T32	≤4yrs

Supplementary File 5: Maternal and area characteristics associated with reaching fidelity targets in each stage of the Family Nurse Partnership programme, for mothers giving birth between April 2010 and January 2018 aged 13-19 at last menstrual period

Presents the data underlying Figure 2 in the main manuscript

Table S5.1 Pregnancy stage

	N mothers (% of all mothers)	N who met pregnancy target (% of group)	Crude relative risk (95% CI)	Adjusted relative risk (95% CI)[a]
**Total**	**27360 (100)**	**16610 (60.7)**	-	-
**Maternal age at birth**				
13-15	1315 (4.8)	930 (70.7)	1.20 (1.16-1.25)	1.12 (1.07-1.17)
16-17	9225 (33.7)	5735 (62.2)	1.05 (1.03-1.07)	1.04 (1.01-1.06)
18-19	13715 (50.1)	8145 (59.4)	1.00 (ref)	1.00 (ref)
20[b]	3105 (11.3)	1800 (58.0)	0.97 (0.94-1.01)	0.97 (0.93-1.01)
**Ethnicity**				
White	23155 (84.6)	14070 (60.8)	1.00 (ref)	1.00 (ref)
South Asian	590 (2.2)	355 (60.2)	1.03 (0.97-1.09)	1.04 (0.98-1.10)
Black	1270 (4.6)	755 (59.4)	1.02 (0.96-1.08)	1.05 (0.99-1.11)
Mixed/other	1465 (5.4)	895 (61.1)	1.04 (0.99-1.08)	1.05 (1.00-1.09)
Unknown	885 (3.2)	535 (60.5)	0.99 (0.93-1.06)	1.00 (0.94-1.06)
**Index of Multiple Deprivation**				
Least deprived	1225 (4.5)	785 (64.1)	1.00 (ref)	1.00 (ref)
2	1990 (7.3)	1245 (62.6)	0.97 (0.93-1.02)	0.99 (0.94-1.03)
3	3595 (13.1)	2235 (62.2)	0.98 (0.93-1.02)	0.99 (0.95-1.03)
4	6890 (25.2)	4140 (60.1)	0.95 (0.91-1.00)	0.97 (0.93-1.01)
Most deprived	13520 (49.4)	8100 (59.9)	0.95 (0.91-1.00)	0.97 (0.93-1.02)
**History of admissions with diagnoses within 2 years prior to 20 weeks gestation**				
Adversity	1950 (7.1)	1285 (65.9)	1.08 (1.04-1.12)	0.96 (0.90-1.03)
Mental health	1065 (4.2)	790 (4.7)	1.10 (1.05-1.14)	1.09 (1.04-1.14)
Chronic condition (any, exc. mental health)	3495 (12.8)	2230 (63.8)	1.05 (1.02-1.08)	0.99 (0.94-1.04)
A&E visits	19075 (69.7)	11555 (60.6)	0.99 (0.97-1.01)	0.98 (0.96-1.00)
Repeated A&E visits	5870 (21.5)	3580 (61.0)	1.00 (0.98-1.03)	0.98 (0.96-1.01)
**Ever in care, prior to 20 weeks gestation**				
No	21930 (80.2)	13160 (60.0)	1.00 (ref)	1.00 (ref)
Yes	2700 (9.9)	1770 (65.7)	1.10 (1.06-1.13)	1.08 (1.04-1.11)
**Ever had recorded child protection plan, prior to 20 weeks gestation**				
No	23150 (84.6)	13960 (60.3)	1.00 (ref)	1.00 (ref)
Yes	1480 (5.4)	970 (65.5)	1.08 (1.03-1.12)	1.01 (0.96-1.05)
**Ever recorded as having special educational needs**				
No	9630 (35.2)	5710 (59.3)	1.00 (ref)	1.00 (ref)
Yes	14870 (54.3)	9150 (61.5)	1.03 (1.01-1.06)	1.02 (1.00-1.05)
**Ever recorded as having free school meals**				
No	8445 (30.9)	5055 (59.9)	1.00 (ref)	1.00 (ref)
Yes	16055 (58.7)	9800 (61.0)	1.02 (1.00-1.04)	1.00 (0.98-1.02)

Table S5.1 continued

	N mothers (% of all mothers)	N who met pregnancy target (% of group)	Crude relative risk (95% CI)	Adjusted relative risk (95% CI)[a]
**Ever excluded, in pupil referral unit or alternative provision, prior to 20 weeks gestation**				
No	15485 (56.6)	9485 (61.3)	1.00 (ref)	1.00 (ref)
Yes	9140 (33.4)	5450 (59.6)	0.97 (0.95-0.99)	0.94 (0.92-0.96)
**Ever recorded as persistently absent in a term, prior to 20 weeks gestation**				
No	11240 (41.1)	6765 (60.2)	1.00 (ref)	1.00 (ref)
Yes	13385 (48.9)	8165 (61.0)	1.02 (1.00-1.04)	0.97 (0.95-0.99)
**Educational attainment, prior to 20 weeks gestation**				
Attempted but did not achieve 5 A*-C GCSEs	17245 (63.0)	10200 (59.1)	1.00 (ref)	1.00 (ref)
Gained 5 A*-C GCSEs	3560 (13.0)	2200 (61.8)	1.04 (1.01-1.07)	1.04 (1.01-1.07)
Too young for GCSEs	3825 (14.0)	2535 (66.3)	1.13 (1.09-1.16)	1.05 (1.01-1.09)
**Number of benefits received at enrolment**				
0	15465 (56.5)	9470 (61.2)	1.00 (ref)	1.00 (ref)
1	6050 (22.1)	3580 (59.2)	0.97 (0.95-1.00)	0.99 (0.97-1.03)
2	3365 (12.3)	2045 (60.8)	1.00 (0.96-1.03)	1.01 (0.97-1.04)
3	1835 (6.7)	1115 (60.8)	1.00 (0.97-1.04)	1.02 (0.98-1.05)
4+	650 (2.4)	400 (61.5)	1.01 (0.95-1.08)	1.01 (0.95-1.08)
**Child in need status at enrolment**				
No	25730 (94.0)	15420 (60.0)	1.00 (ref)	1.00 (ref)
Yes	1630 (6.0)	1190 (72.7)	1.21 (1.17-1.25)	1.17 (1.14-1.21)
**Child protection plan at enrolment**				
No	26580 (97.1)	16020 (60.3)	1.00 (ref)	1.00 (ref)
Yes	780 (2.9)	590 (75.6)	1.26 (1.20-1.32)	1.20 (1.14-1.26)
**% of visits with partner present, median (IQR)**	13 (2-34.4)	14.3 (2.5-35.7)	1.00 (1.00-1.00)	1.00 (1.00-1.00)
**% of visits with parent present, median (IQR)**	5.9 (0-19.4)	6.4 (0-20)	1.00 (1.00-1.00)	1.00 (1.00-1.00)
**Area characteristics**				
**IMD 2015 quintiles**				
Least deprived	6875 (25.1)	4025 (58.5)	1.00 (ref)	1.00 (ref)
2	6845 (25.0)	4115 (60.1)	1.02 (0.95-1.09)	1.01 (0.94-1.08)
3	4625 (16.9)	2855 (61.7)	1.06 (0.99-1.13)	1.05 (0.98-1.12)
4	5945 (21.7)	3560 (59.9)	1.04 (0.97-1.12)	1.03 (0.96-1.11)
Most deprived	3070 (11.2)	2050 (66.8)	1.11 (1.04-1.20)	1.10 (1.03-1.18)
**% of eligible mothers enrolled in FNP**				
<=21%	9945 (36.3)	6050 (60.8)	1.00 (ref)	1.00 (ref)
21-28%	7440 (27.2)	4550 (61.2)	0.98 (0.89-1.08)	0.98 (0.90-1.08)
28-36%	5535 (20.2)	3190 (57.6)	0.94 (0.86-1.03)	0.97 (0.89-1.07)
36-67%	4435 (16.2)	2820 (63.6)	1.02 (0.93-1.11)	1.03 (0.94-1.12)
**% of the population who are Black, median (IQR)**	1.5 (.6-6)	1.5 (.6-3.9)	0.99 (0.99-1.00)	1.00 (0.99-1.00)

[a] Adjusted models included all variables in the table

[b] Includes only mothers aged 19 at last menstrual period

CI: confidence interval; GCSE: General Certificate of Secondary Education; IQR: interquartile range; IMD: Index of Multiple Deprivation

Note: numbers have been rounded to the nearest 5 and cell sizes <10 have been suppressed, in accordance with NHS Digital's and DfE's statistical disclosure rules for sub-national analyses

Table S5.2 Infancy stage

	N mothers (% of all mothers)	N who met infancy target (% of group)	Crude relative risk (95% CI)	Adjusted relative risk (95% CI)[a]
**Total**	**25635 (100)**	**16845 (65.7)**	-	-
**Met pregnancy target**				
No	9405 (36.7)	4420 (47.0)	1.00 (ref)	1.00 (ref)
Yes	16230 (63.3)	12425 (76.6)	1.58 (1.53-1.64)	1.56 (1.50-1.61)
**Maternal age at birth**				
13-15	1270 (5.0)	935 (73.6)	1.13 (1.08-1.17)	1.07 (1.03-1.12)
16-17	8695 (33.9)	5730 (65.9)	1.01 (0.99-1.03)	1.01 (0.98-1.03)
18-19	12760 (49.8)	8330 (65.3)	1.00 (ref)	1.00 (ref)
20[b]	2905 (11.3)	1850 (63.7)	0.98 (0.95-1.01)	0.98 (0.95-1.01)
**Ethnicity**				
White	21650 (84.5)	14425 (66.6)	1.00 (ref)	1.00 (ref)
South Asian	545 (2.1)	350 (64.2)	1.01 (0.93-1.09)	1.00 (0.92-1.08)
Black	1220 (4.8)	695 (57.0)	0.92 (0.86-0.98)	0.91 (0.85-0.97)
Mixed/other	1380 (5.4)	835 (60.5)	0.95 (0.90-0.99)	0.94 (0.90-0.98)
Unknown	835 (3.3)	540 (64.7)	0.96 (0.90-1.02)	0.97 (0.91-1.03)
**Index of Multiple Deprivation**				
Least deprived	1135 (4.4)	770 (67.8)	1.00 (ref)	1.00 (ref)
2	1855 (7.2)	1275 (68.7)	1.02 (0.96-1.09)	1.04 (0.98-1.10)
3	3335 (13.0)	2265 (67.9)	1.02 (0.97-1.07)	1.04 (0.99-1.09)
4	6475 (25.3)	4260 (65.8)	1.02 (0.96-1.08)	1.05 (0.99-1.10)
Most deprived	12700 (49.5)	8175 (64.4)	1.01 (0.96-1.07)	1.04 (0.99-1.09)
**History of admissions with diagnoses within 2 years prior to 20 weeks gestation**				
Adversity	1835 (7.2)	1295 (70.6)	1.07 (1.03-1.10)	0.96 (0.91-1.10)
Mental health	1065 (4.2)	790 (4.7)	1.10 (1.05-1.14)	1.07 (1.02-1.13)
Chronic conditions (any, excl. mental health)	3295 (12.9)	2285 (69.3)	1.05 (1.03-1.08)	1.00 (0.97-1.04)
A&E visits	17835 (69.6)	11780 (66.0)	1.02 (1.00-1.04)	1.01 (0.99-1.03)
Repeated A&E visits	5490 (21.4)	3720 (67.8)	1.05 (1.02-1.07)	1.03 (1.01-1.05)
**Ever in care, prior to 20 weeks gestation**				
No	20535 (80.1)	13395 (65.2)	1.00 (ref)	1.00 (ref)
Yes	2535 (9.9)	1810 (71.4)	1.10 (1.06-1.14)	1.05 (1.01-1.08)
**Ever had recorded child protection plan, prior to 20 weeks gestation**				
No	21685 (84.6)	14215 (65.6)	1.00 (ref)	1.00 (ref)
Yes	1390 (5.4)	990 (71.2)	1.08 (1.04-1.12)	1.03 (1.00-1.07)
**Ever recorded as having special educational needs, prior to 20 weeks gestation**				
No	8995 (35.1)	5765 (64.1)	1.00 (ref)	1.00 (ref)
Yes	13950 (54.4)	9365 (67.1)	1.04 (1.02-1.06)	1.04 (1.01-1.05)
**Ever recorded as having free school meals**				
No	7915 (30.9)	5190 (65.6)	1.00 (ref)	1.00 (ref)
Yes	15035 (58.7)	9940 (66.1)	1.02 (1.00-1.04)	1.00 (0.98-1.02)

Table S5.2 continued

	N mothers (% of all mothers)	N who met infancy target (% of group)	Crude relative risk (95% CI)	Adjusted relative risk (95% CI)[a]
**Ever excluded, in pupil referral unit or alternative provision, prior to 20 weeks gestation**				
No	14530 (56.7)	9640 (66.3)	1.00 (ref)	1.00 (ref)
Yes	8545 (33.3)	5565 (65.1)	0.98 (0.96-1.00)	0.97 (0.95-0.98)
**Ever recorded as persistently absent in a term, prior to 20 weeks gestation**				
No	10490 (40.9)	6945 (66.2)	1.00 (ref)	1.00 (ref)
Yes	12585 (49.1)	8260 (65.6)	0.99 (0.98-1.01)	0.97 (0.95-0.99)
**Educational attainment, prior to 20 weeks gestation**				
Attempted but did not achieve 5 A*-C GCSEs	16115 (62.9)	10480 (65.0)	1.00 (ref)	1.00 (ref)
Gained 5 A*-C GCSEs	3310 (12.9)	2215 (66.9)	1.03 (1.00-1.05)	1.03 (1.00-1.05)
Too young for GCSEs	3645 (14.2)	2510 (68.9)	1.06 (1.03-1.09)	1.01 (0.98-1.04)
**Number of benefits received at enrolment**				
0	14475 (56.5)	9495 (65.6)	1.00 (ref)	1.00 (ref)
1	5660 (22.1)	3685 (65.1)	1.00 (0.98-1.03)	1.00 (0.98-1.03)
2	3160 (12.3)	2110 (66.8)	1.02 (0.99-1.05)	1.01 (0.98-1.04)
3	1725 (6.7)	1140 (66.1)	1.02 (0.99-1.06)	1.00 (0.97-1.04)
4+	615 (2.4)	410 (66.7)	1.03 (0.98-1.10)	1.01 (0.95-1.07)
**Child in need status at enrolment**				
No	24080 (93.9)	15680 (65.1)	1.00 (ref)	1.00 (ref)
Yes	1560 (6.1)	1170 (74.7)	1.14 (1.10-1.19)	1.07 (1.04-1.11)
**Child protection plan at enrolment**				
No	24880 (97.1)	16250 (65.3)	1.00 (ref)	1.00 (ref)
Yes	755 (2.9)	595 (78.8)	1.23 (1.17-1.29)	1.11 (1.07-1.17)
**% of visits with partner present, median (IQR)**	13.3 (2.4-34.1)	14.3 (3.2-35.3)	1.00 (1.00-1.00)	1.00 (1.00-1.00)
**% of visits with parent present, median (IQR)**	6.3 (0-19.2)	6.3 (0-19.4)	1.00 (1.00-1.00)	1.00 (1.00-1.00)
**Area characteristics**				
**IMD 2015 quintiles**				
Least deprived	6440 (25.1)	3925 (60.9)	1.00 (ref)	1.00 (ref)
2	6385 (24.9)	4100 (64.2)	1.04 (0.96-1.12)	1.03 (0.97-1.10)
3	4365 (17.0)	2925 (67.0)	1.09 (1.01-1.17)	1.07 (1.01-1.14)
4	5570 (21.7)	3755 (67.4)	1.08 (1.01-1.15)	1.08 (1.01-1.14)
Most deprived	2870 (11.2)	2140 (74.6)	1.11 (1.02-1.20)	1.12 (1.04-1.20)
**% of eligible mothers enrolled in FNP**				
<=21%	9300 (36.3)	6195 (66.6)	1.00 (ref)	1.00 (ref)
21-28%	6960 (27.2)	4640 (66.7)	0.98 (0.89-1.07)	0.98 (0.92-1.05)
28-36%	5200 (20.3)	3205 (61.6)	0.92 (0.84-1.00)	0.95 (0.89-1.02)
36-67%	4170 (16.3)	2805 (67.3)	0.99 (0.91-1.08)	0.99 (0.92-1.05)
**% of the population who are Black, median (IQR)**	1.6 (.6-6)	1.4 (.6-3.9)	0.99 (0.99-1.00)	1.00 (0.99-1.00)

[a] Adjusted models included all variables in the table

[b] Includes only mothers aged 19 at last menstrual period

CI: confidence interval; GCSE: General Certificate of Secondary Education; IQR: interquartile range; IMD: Index of Multiple Deprivation

Note: numbers have been rounded to the nearest 5 and cell sizes <10 have been suppressed, in accordance with NHS Digital's and DfE's statistical disclosure rules for sub-national analyses

Table S5.3 Toddlerhood stage

	N mothers (% of all mothers)	N who met toddlerhood target (% of group)	Crude relative risk (95% CI)	Adjusted relative risk (95% CI)[a]
**Total**	**19655 (100)**	**11900 (60.5)**	-	-
**Met pregnancy target**				
No	6835 (34.8)	3300 (48.3)	1.00 (ref)	1.00 (ref)
Yes	12820 (65.2)	8600 (67.1)	1.34 (1.30-1.39)	1.16 (1.13-1.19)
**Met infancy target**				
No	5805 (29.5)	1945 (33.5)	1.00 (ref)	1.00 (ref)
Yes	13850 (70.5)	9955 (71.9)	2.08 (1.98-2.19)	1.98 (1.90-2.07)
**Maternal age at birth**				
13-15	970 (4.9)	595 (61.3)	1.02 (0.97-1.07)	0.96 (0.91-1.02)
16-17	6675 (34.0)	4035 (60.4)	1.00 (0.97-1.02)	0.99 (0.96-1.02)
18-19	9865 (50.2)	5995 (60.8)	1.00 (ref)	1.00 (ref)
20[b]	2145 (10.9)	1275 (59.4)	0.98 (0.94-1.01)	0.99 (0.96-1.03)
**Ethnicity**				
White	16610 (84.5)	10235 (61.6)	1.00 (ref)	1.00 (ref)
South Asian	410 (2.1)	255 (62.2)	0.97 (0.91-1.05)	1.01 (0.94-1.08)
Black	950 (4.8)	470 (49.5)	0.86 (0.80-0.93)	0.88 (0.81-0.94)
Mixed/other	1035 (5.3)	550 (53.1)	0.89 (0.83-0.94)	0.91 (0.85-0.96)
Unknown	645 (3.3)	390 (60.5)	0.95 (0.88-1.03)	1.00 (0.94-1.06)
**Index of Multiple Deprivation**				
Least deprived	900 (4.6)	550 (61.1)	1.00 (ref)	1.00 (ref)
2	1465 (7.5)	875 (59.7)	0.99 (0.92-1.06)	0.97 (0.92-1.03)
3	2585 (13.2)	1580 (61.1)	1.02 (0.97-1.08)	1.00 (0.96-1.05)
4	4915 (25.0)	2955 (60.1)	1.04 (0.97-1.10)	1.01 (0.96-0.16)
Most deprived	9695 (49.3)	5860 (60.4)	1.05 (0.99-1.11)	1.01 (0.97-1.06)
**History of admissions with diagnoses within 2 years prior to 20 weeks gestation**				
Adversity	1350 (6.9)	865 (64.1)	1.05 (1.00-1.09)	0.98 (0.91-1.05)
Mental health	775 (4.0)	615 (4.4)	1.08 (1.02-1.14)	1.04 (0.95-1.13)
Chronic condition (any, exc. mental health)	2445 (12.4)	1545 (63.2)	1.04 (1.01-1.08)	1.00 (0.96-1.05)
A&E visits	13520 (68.8)	8230 (60.9)	1.02 (1.00-1.04)	1.00 (0.98-1.03)
Repeated A&E visits	4015 (20.4)	2530 (63.0)	1.06 (1.03-1.09)	1.05 (1.03-1.08)
**Ever in care, prior to 20 weeks gestation**				
No	16060 (81.7)	9680 (60.2)	1.00 (ref)	1.00 (ref)
Yes	1710 (8.7)	1090 (63.7)	1.06 (1.02-1.11)	1.01 (0.96-1.05)
**Ever had recorded child protection plan, prior to 20 weeks gestation**				
No	16840 (85.7)	10190 (60.6)	1.00 (ref)	1.00 (ref)
Yes	930 (4.7)	570 (61.3)	1.01 (0.96-1.06)	1.01 (0.96-1.06)
**Ever recorded as having special educational needs, prior to 20 weeks gestation**				
No	7105 (36.1)	4190 (59.0)	1.00 (ref)	1.00 (ref)
Yes	10560 (53.7)	6520 (61.7)	1.04 (1.01-1.07)	1.03 (1.00-1.06)

Table S5.3 continued

	N mothers (% of all mothers)	N who met toddlerhood target (% of group)	Crude relative risk (95% CI)	Adjusted relative risk (95% CI)[a]
**Ever recorded as having free school meals, prior to 20 weeks gestation**				
No	6330 (32.2)	3760 (59.4)	1.00 (ref)	1.00 (ref)
Yes	11340 (57.7)	6950 (61.3)	1.04 (1.01-1.07)	1.05 (1.03-1.07)
**Ever excluded, in pupil referral unit or alternative provision, prior to 20 weeks gestation**				
No	11335 (57.7)	6895 (60.8)	1.00 (ref)	-
Yes	6435 (32.7)	3870 (60.1)	0.98 (0.96-1.01)	0.98 (0.96-1.00)
**Ever recorded as persistently absent in a term, prior to 20 weeks gestation**				
No	8025 (40.8)	4890 (60.9)	1.00 (ref)	1.00 (ref)
Yes	9745 (49.6)	5880 (60.3)	0.99 (0.97-1.02)	0.98 (0.96-1.01)
**Educational attainment, prior to 20 weeks gestation**				
Attempted but did not achieve 5 A*-C GCSEs	12340 (62.8)	7530 (61.0)	1.00 (ref)	1.00 (ref)
Gained 5 A*-C GCSEs	2640 (13.4)	1525 (57.8)	0.95 (0.92-0.98)	0.97 (0.94-1.00)
**Number of benefits received at enrolment**				
0	11115 (56.6)	6525 (58.7)	1.00 (ref)	1.00 (ref)
1	4350 (22.1)	2755 (63.3)	1.09 (1.06-1.12)	1.04 (1.01-1.07)
2	2400 (12.2)	1470 (61.3)	1.04 (1.01-1.08)	1.00 (0.97-1.04)
3	1305 (6.6)	840 (64.4)	1.10 (1.05-1.16)	1.04 (1.00-1.10)
4+	485 (2.5)	315 (64.9)	1.11 (1.04-1.18)	1.03 (0.97-1.09)
**Child in need status at enrolment**				
No	18560 (94.4)	11180 (60.2)	1.00 (ref)	1.00 (ref)
Yes	1095 (5.6)	720 (65.8)	1.08 (1.03-1.14)	1.01 (0.97-1.06)
**Child protection plan at enrolment**				
No	19155 (97.5)	11560 (60.3)	1.00 (ref)	1.00 (ref)
Yes	500 (2.5)	340 (68.0)	1.13 (1.06-1.21)	1.01 (0.95-1.07)
**% of visits with partner present, median (IQR)**	13.8 (3-33.3)	15 (3.8-35)	1.00 (1.00-1.00)	1.00 (1.00-1.00)
**% of visits with parent present, median (IQR)**	6.3 (1.5-18.6)	6.4 (1.8-18.8)	1.00 (1.00-1.00)	1.00 (1.00-1.00)
**Area characteristics**				
**IMD 2015 quintiles**				
Least deprived	4600 (23.4)	2670 (58.0)	1.00 (ref)	1.00 (ref)
2	5065 (25.8)	3035 (59.9)	0.98 (0.90-1.08)	1.00 (0.93-1.07)
3	3360 (17.1)	2085 (62.1)	1.00 (0.93-1.08)	1.01 (0.94-1.07)
4	4355 (22.2)	2540 (58.3)	0.97 (0.90-1.04)	0.98 (0.91-1.05)
Most deprived	2275 (11.6)	1570 (69.0)	1.05 (0.96-1.15)	1.08 (1.01-1.16)
**% of eligible mothers enrolled in FNP**				
<=21%	7120 (36.2)	4395 (61.7)	1.00 (ref)	1.00 (ref)
21-28%	5305 (27.0)	3335 (62.9)	0.97 (0.88-1.08)	1.03 (0.95-1.11)
28-36%	4025 (20.5)	2190 (54.4)	0.84 (0.75-0.94)	0.95 (0.88-1.04)
36-67%	3205 (16.3)	1980 (61.8)	0.95 (0.87-1.04)	1.03 (0.96-1.10)

Table S5.3 continued

	N mothers (% of all mothers)	N who met toddlerhood target (% of group)	Crude relative risk (95% CI)	Adjusted relative risk (95% CI)[a]
**Rate of premature births (<37w), per 1000 births, median (IQR)**	79.3 (74.2-84)	79 (73.1-83.7)	1.00 (0.99-1.00)	1.00 (0.99-1.00)
**Rate of referrals to children's social care, per 10,000 <18s, median (IQR)**	511.8 (436.5-691.3)	511.8 (431.3-691.3)	1.00 (1.00-1.00)	1.00 (1.00-1.00)

[a] Adjusted models included all variables in the table

[b] Includes only mothers aged 19 at last menstrual period

CI: confidence interval; GCSE: General Certificate of Secondary Education; IQR: interquartile range; IMD: Index of Multiple Deprivation

Note: numbers have been rounded to the nearest 5 and cell sizes <10 have been suppressed, in accordance with NHS Digital's and DfE's statistical disclosure rules for sub-national analyses

Supplementary File 6: Sensitivity analysis using multiple imputation for the association between maternal and area characteristics and fidelity targets in the pregnancy stage of the Family Nurse Partnership programme, for mothers giving birth between April 2010 and January 2018 aged 13-19 at last menstrual period

	N mothers (% of all mothers)	N who met pregnancy target (% of group)	Adjusted relative risk[a] (95% CI)	Adjusted relative risk[a] (95% CI) Multiple imputation
**Total**	**27360 (100)**	**16610 (60.7)**	**-**	-
**Maternal age at birth**				
13-15	1315 (4.8)	930 (70.7)	1.12 (1.07-1.17)	1.11 (1.06-1.17)
16-17	9225 (33.7)	5735 (62.2)	1.04 (1.01-1.06)	1.04 (1.01-1.06)
18-19	13720 (50.1)	8145 (59.4)	1.00 (ref)	1.00 (ref)
20[b]	3105 (11.3)	1800 (58.0)	0.97 (0.93-1.01)	0.97 (0.93-1.00)
**Ethnicity**				
White	23155 (84.6)	14070 (60.8)	1.00 (ref)	1.00 (ref)
South Asian	590 (2.2)	355 (60.2)	1.04 (0.98-1.10)	0.99 (0.93-1.06)
Black	1270 (4.6)	755 (59.4)	1.05 (0.99-1.11)	1.00 (0.95-1.05)
Mixed/other	1465 (5.4)	895 (61.1)	1.05 (1.00-1.09)	1.02 (0.98-1.06)
Unknown	885 (3.2)	535 (60.5)	1.00 (0.94-1.06)	1.00 (0.94-1.05)
**Index of Multiple Deprivation**				
Least deprived	1225 (4.5)	785 (64.1)	1.00 (ref)	1.00 (ref)
2	1990 (7.3)	1245 (62.6)	0.99 (0.94-1.03)	0.99 (0.94-1.05)
3	3595 (13.1)	2235 (62.2)	0.99 (0.95-1.03)	1.00 (0.95-1.05)
4	6890 (25.2)	4140 (60.1)	0.97 (0.93-1.01)	0.97 (0.93-1.02)
Most deprived	13520 (49.4)	8100 (59.9)	0.97 (0.93-1.02)	0.98 (0.93-1.03)
**History of admissions with diagnoses within 2 years prior to 20 weeks gestation**				
Adversity	1950 (7.1)	1285 (65.9)	0.96 (0.90-1.03)	1.03 (0.98-1.09)
Mental health	1065 (4.2)	790 (4.7)	1.09 (1.04-1.14)	1.11 (1.06-1.17)
Chronic condition (any, exc. mental health)	3495 (12.8)	2230 (63.8)	0.99 (0.94-1.04)	1.00 (0.96-1.05)
A&E visits	19075 (69.7)	11555 (60.6)	0.98 (0.96-1.00)	0.98 (0.96-1.01)
Repeated A&E visits	5870 (21.5)	3580 (61.0)	0.98 (0.96-1.01)	0.98 (0.96-1.01)
**Ever in care, prior to 20 weeks gestation**				
No	21930 (80.2)	13160 (60.0)	1.00 (ref)	1.00 (ref)
Yes	2700 (9.9)	1770 (65.7)	1.08 (1.04-1.11)	1.07 (1.04-1.11)
**Ever had recorded child protection plan, prior to 20 weeks gestation**				
No	23150 (84.6)	13960 (60.3)	1.00 (ref)	1.00 (ref)
Yes	1480 (5.4)	970 (65.5)	1.01 (0.96-1.05)	1.01 (0.97-1.05)
**Ever recorded as having special educational needs, prior to 20 weeks gestation**				
No	9630 (35.2)	5710 (59.3)	1.00 (ref)	1.00 (ref)
Yes	14870 (54.3)	9150 (61.5)	1.02 (1.00-1.05)	1.03 (1.00-1.05)
**Ever recorded as having free school meals, prior to 20 weeks gestation**				
No	8445 (30.9)	5055 (59.9)	1.00 (ref)	1.00 (ref)
Yes	16055 (58.7)	9800 (61.0)	1.00 (0.98-1.02)	1.00 (0.98-1.02)

Supplementary table 6 continued

	N mothers (% of all mothers)	N who met pregnancy target (% of group)	Adjusted relative risk[a] (95% CI)	Adjusted relative risk[a] (95% CI) Multiple imputation
**Ever excluded, in pupil referral unit or alternative provision, prior to 20 weeks gestation**				
No	15490 (56.6)	9485 (61.2)	1.00 (ref)	1.00 (ref)
Yes	9140 (33.4)	5450 (59.6)	0.94 (0.92-0.96)	0.94 (0.92-0.96)
**Ever recorded as persistently absent in a term, prior to 20 weeks gestation**				
No	11245 (41.1)	6765 (60.2)	1.00 (ref)	1.00 (ref)
Yes	13385 (48.9)	8165 (61.0)	0.97 (0.95-0.99)	0.96 (0.94-0.99)
**Educational attainment, prior to 20 weeks gestation**				
Attempted but did not achieve 5 A*-C GCSEs	17245 (63.0)	10200 (59.1)	1.00 (ref)	1.00 (ref)
Gained 5 A*-C GCSEs	3560 (13.0)	2200 (61.8)	1.04 (1.01-1.07)	1.05 (1.02-1.08)
**Number of benefits received at enrolment**				
0	15465 (56.5)	9470 (61.2)	1.00 (ref)	1.00 (ref)
1	6050 (22.1)	3580 (59.2)	0.99 (0.97-1.03)	0.99 (0.97-1.02)
2	3365 (12.3)	2045 (60.8)	1.01 (0.97-1.04)	1.00 (0.97-1.03)
3	1835 (6.7)	1115 (60.8)	1.02 (0.98-1.05)	1.01 (0.97-1.05)
4+	650 (2.4)	400 (61.5)	1.01 (0.95-1.08)	1.00 (0.94-1.07)
**Child in need status at enrolment**				
No	25730 (94.0)	15425 (59.9)	1.00 (ref)	1.00 (ref)
Yes	1630 (6.0)	1185 (72.7)	1.17 (1.14-1.21)	1.17 (1.13-1.21)
**Child protection plan at enrolment**				
No	26580 (97.1)	16020 (60.3)	1.00 (ref)	1.00 (ref)
Yes	780 (2.9)	590 (75.6)	1.20 (1.14-1.26)	1.20 (1.15-1.25)
**% of visits with partner present, median(IQR)**	13 (2-34.4)	14.3 (2.5-35.7)	1.00 (1.00-1.00)	1.00 (1.00-1.00)
**% of visits with parent present, median (IQR)**	5.9 (0-19.4)	6.4 (0-20)	1.00 (1.00-1.00)	1.00 (1.00-1.00)
**Area characteristics**				
**IMD 2015 quintiles**				
Least deprived	6875 (25.1)	4025 (58.5)	1.00 (ref)	1.00 (ref)
2	6845 (25.0)	4115 (60.1)	1.01 (0.94-1.08)	1.01 (0.99-1.04)
3	4625 (16.9)	2855 (61.7)	1.05 (0.98-1.12)	1.05 (1.01-1.08)
4	5945 (21.7)	3560 (59.9)	1.03 (0.96-1.11)	1.00 (0.97-1.04)
Most deprived	3070 (11.2)	2050 (66.8)	1.10 (1.03-1.18)	1.12 (1.08-1.16)
**% of eligible mothers enrolled in FNP**	24 (19-31)	24 (19-33)	1.00 (1.00-1.00)	1.00 (1.00-1.00)

[a] Adjusted models included all variables in the table

[b] Includes only mothers aged 19 at last menstrual period

CI: confidence interval; GCSE: General Certificate of Secondary Education; IQR: interquartile range; IMD: Index of Multiple Deprivation

Note: numbers have been rounded to the nearest 5 and cell sizes <10 have been suppressed, in accordance with NHS Digital's and DfE's statistical disclosure rules for sub-national analyses
